# Two New Derivatives of 2, 5-Dihydroxyphenylacetic Acid from the Kernel of *Entada phaseoloides*

**DOI:** 10.3390/molecules18021477

**Published:** 2013-01-25

**Authors:** Lin Chen, Yong Zhang, Gang Ding, Mingyu Ba, Ying Guo, Zhongmei Zou

**Affiliations:** 1 Institute of Medicinal Plant Development, Chinese Academy of Medical Sciences and Peking Union Medical College, Beijing 100193, China; E-Mails: cllg@live.cn (L.C.); pope95@163.com (Y.Z.); gding@implad.ac.cn (G.D.); 2 Beijing Key Laboratory of New Drug Mechanisms and Pharmacological Evaluation Study (No. BZ0150), Institute of Material Medica, Chinese Academy of Medical Sciences and Peking Union Medical College, Beijing 100050, China; E-Mail: bamingyu@imm.ac.cn

**Keywords:** *Entada phaseoloides*, Leguminosae, 2,5-dihydroxyphenylacetic acid, HIV-1

## Abstract

Two new aromatic compounds, butyl 2,5-dihydroxyphenylacetate (**1**) and butyl 2-O-*β*-D-glucopyranosyloxy-5-dihydroxyphenylacetate (**2**), together with three known ones, methyl 2,5-dihydroxyphenylacetate (**3**), ethyl 2,5-dihydroxyphenylacetate (**4**) and 2-O-*β*-D-glucopyranosyloxy-5-hydroxyphenylacetic acid (**5**), were isolated from the EtOH extract of the kernel of *Entada phaseoloides*. The structures of the new compounds were elucidated by MS and NMR experiments. Compounds **1**, **3** and **4** displayed potent inhibitory activities against HIV-1 replication, with EC_50_ values of 9.80 *μ*M, 11.70 *μ*M and 9.93 *μ*M, respectively.

## 1. Introduction

*Entada phaseoloides* (L.) Merr. is the sole species of *Entada* genus (Leguminosae), widely distributed in south China, esp. in Yunnan and Hainan provinces. The kernel of *E. phaseoloides* has been commonly used as a herbal medicine by the Dai nationality for the treatment of hemostasis and detoxification [1]. Some investigations suggested that it had antidiabetic [2], anti-inflammatory [3], and molluscicidal activities [4]. In 1955, Barua first obtained a triterpene acid from its kernel [5]. After that, more compounds from this plant were reported, such as phenylacetic acid esters [6,7], triterpene saponins [8], phenolic acids [9,10], chalcone glycosides [11] and sulfur-containing amides [12,13]. As one of the components in Qi-wei Ke-Teng-Zi Wan [14], a famous formula of medicines used in the Dai nationality, the active constituents of the kernel of *E. phaseoloides* are still unknown. Herein we report the isolation, structure elucidation, and anti-HIV activity of five aromatic compounds from the kernel of *Entada phaseoloides*. 

## 2. Results and Discussion

**Figure 1:**
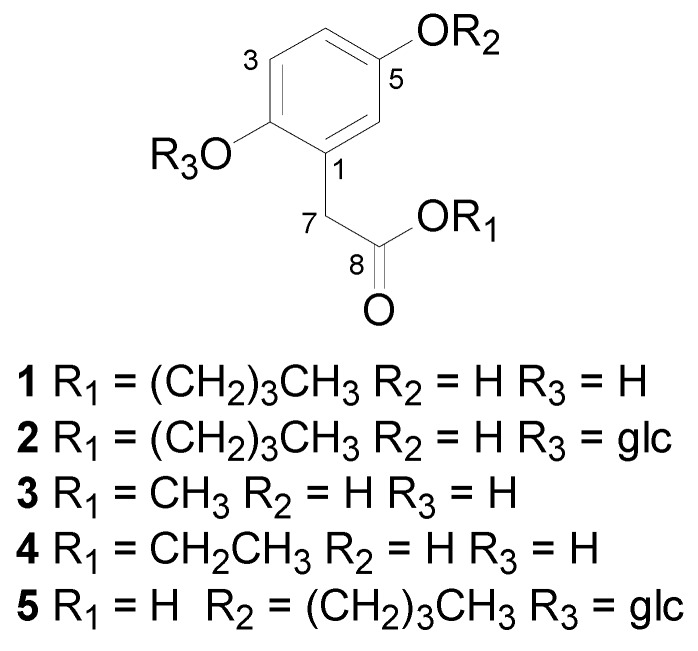
Structures of compounds **1**–**5**.

The EtOH extract from air-dried kernels (7 kg) of *E. phaseoloides* was extracted with petroleum ether, EtOAc, and *n*-BuOH. The EtOAc extract was separated by repeated column chromatography to give five aromatic compounds (Figure 1) including two new ones (compounds **1**,**2**). The known compounds were readily identified as methyl 2,5-dihydroxyphenylacetate (**3**) [1], ethyl 2,5-dihydroxy-phenylacetate (**4**) [6] and 2-O-*β*-D-glucopyranosyloxy-5-hydroxyphenylacetic acid (**5**) [6] by comparison of their spectroscopic data with published values.

Compound **1** was obtained as a white crystalline solid. The molecular formula was determined as C_12_H_16_O_4_ (five degrees of unsaturation) on the basis of Its TOF-ESI-MS at *m/z* 247.0963 [M+Na]^+^. The IR spectrum of **1** showed absorption bands at 3390 (OH), 1722 (C=O) cm^−1^. The ^1^H-NMR data (Table 1) of **1** revealed similar structural features as those of **3**, except for the additional signals of the butyl group at 0.92 (3H, t, *J* = 7.5 Hz), 1.64 (2H, m), 1.37 (2H, m), 4.14 (2H, t, *J* = 6.5 Hz). This implied that the methyl in **3** was replaced by the butyl in compound **1**. HMBC correlation of 9-H with C-8 showed the butyl group was connected to the ester bond. Hence, **1** was elucidated as butyl 2,5-dihydroxyphenylacetate.

Compound **2** was obtained as a white crystalline solid and displayed similar UV and IR profiles to those of **1**. The molecular formula was determined to be C_18_H_26_O_9_ (six degrees of unsaturation) by analysis of its TOF-ESI-MS at *m/z* 409.1463 [M+Na]^+^. Compared with the NMR data with those of **1**, it revealed that **2** possessed similar units with those of **1** except that an additional glucose moiety [*δ*_H_ 4.69 (1H, d, *J* = 7.5 Hz), 3.41 (1, dd, *J* = 3.0, 6.0 Hz), 3.37 (1H, m), 3.39 (1H, m), 3.45 (1H, m), 3.87 (1H, dd, *J* = 1.5, 12.5 Hz), 3.68 (1H, d, *J* = 12.5 Hz)] was present in **2**. HMBC correlation from 1’-H to C-2 demonstrated the glucose was connected to C-2. Upon acid hydrolysis, **2** afforded D-glucose, which was identified by co-TLC with authentic samples. The *β* configuration of the glucopyranose was confirmed by the large coupling constant (*J* = 7.5 Hz) of its anomeric proton. Thus, the structure of **2** was established as butyl 2-O-*β*-D-glucopyranosyloxy-5-dihydroxyphenylacetate.

All isolated compounds were evaluated for anti-HIV activity against VSVG/HIV pseudotyped virus using zidovudine as the positive control. Compounds 1, 3 and 4 exhibited inhibitory activities against HIV-1 replication with EC_50_ values of 9.80 *μ*M, 11.70 *μ*M and 9.93 *μ*M, respectively, while the EC_50_ values of zidovudine was 11.70 *n*M. However, compounds 2 and 5 did not show inhibition at the concentration of 10 *μ*M.

## 3. Experimental

### 3.1. General 

NMR spectra were measured on a Bruker AM 500 NMR spectrometer as the internal reference and chemical shifts are expressed in ppm. TOF-ESI-MS spectra were measured on a Waters Synapt G2 mass spectrometer. EIMS data were recorded on a Zabspec E mass spectrometer. IR spectra were recorded on a Shimadzu FTIR-8400S spectrophotometer. UV spectra were run on a Shimadzu UV-2550 UV/Vis spectrophotometer. TLC was performed on silica gel GF254 (10–40 μm; Qingdao Marine Chemical, Inc., Qingdao, China). Column chromatography was performed on silica gel (100–200 or 200–300 mesh; Qingdao Marine Chemical, Inc.).

### 3.2. Plant Material

The kernal of *Entada phaseoloides* was collected from Xishuangbanna, Yunnan Province, in January, 2010. The sample was identified by Professor Peng Chaozhong, and a voucher specimen (No. 20100128) has been deposited in the herbarium of the Institute of Medicinal Plant Development, Chinese Academy of Medical Sciences and Peking Union Medical College.

### 3.3. Extraction and Isolation 

The air-dried kernels (7 kg) of *E. phaseoloides* were extracted with 95% and 50% EtOH (10 L) by reflux for 3 times. After removal of the solvent, the aqueous residue was partitioned successively with petroleum ether, EtOAc, and *n*-BuOH. The EtOAc extract (23.3 g) was fractionated by silica gel column chromatography eluted with CH_2_Cl_2_/EtOAc (100:0–5:1) to give six fractions A–G. Fraction C (12.2 g) was subjected to silica gel column chromatography, eluted with petroleum ether/EtOAc (60:1–5:1) to give five fractions (C1–C5). Fraction C2 (4.5 g) was further separated over silica gel column chromatography and the material was eluted using petroleum ether/acetone (40:1–5:1) to afford five fractions (C21–C25). Fraction C22 (924 mg) was chromatographed on Sephadex LH-20 to yield **3** (20 mg). Fraction C23 (812 mg) was chromatographed on Sephadex LH-20 to yield **1** (20 mg) and **4** (8 mg). Fraction C24 (523 mg) was chromatographed on Sephadex LH-20 to yield **2** (15 mg) and **5** (25 mg).

### 3.4. Spectral Data

*Butyl 2, 5-dihydroxyphenylacetate* (**1**) White crystals, mp 135–136 °C; UV (MeOH) *λ*_max_ (log*ε*) 204, 296 nm; IR (KBr)*ν*_max_ 3390 (OH), 1722 (C=O), 1506, 1477, 953, cm^−1^; TOF-ESI-MS *m/z* 247.0963 [M+Na]^+^ (calcd 247.0948 for C_12_H_16_O_4_Na); EI-MS *m/z* (%) 224 [M]^+^ (10), 150 (74), 122 (100), 94 (49), 56 (30), 41 (59); ^1^H-NMR and ^13^C-NMR data see Table 1.

*Butyl 2-O-β-D-glucopyranosyloxy-5-dihydroxyphenylacetate* (**2**) White crystals, mp 135–136 °C; UV (MeOH) *λ*_max_ (log*ε*) 203, 224, 289 nm; IR (KBr) *ν*_max_ 3380 (OH), 1717 (C=O), 1507, 1476, 990 cm^−1^; TOF-ESI-MS *m*/*z* 409.1476 [M+Na]^+^ (calcd 409.1463 for C_18_H_26_O_9_Na); EI-MS*m/z* (%) 224 [M-glc]^+^ (64), 150 (100), 122 (42), 94 (8), 85 (12), 73 (14), 57 (20), 41 (21); ^1^H-NMR and ^13^C-NMR data see Table 1.

### 3.5. Anti-HIV Activity Assay 

Production of VSV-G/HIV pseudovirions: Human embryonic kidney 293T cells were transiently co-transected with 3 μg vesicular stomatitis virus glycoprotein (VSV-G) plasmid and 8 μg Env-deficient HIV vector (pNL4-3-Luc-R^−^E^−^) in 100-mm plates by a standard Ca_3_(PO_4_)_2_ protocol. Sixteen h post-transfection, cells were washed by PBS, then added 10 mL fresh medium into each plate. Forty-eight h post-transfection, the supernatants, containing pseudotyped virions (VSVG/HIV), were collected and filtered through a 0.45 μm filter. Virions were quantified by p24 concentrations which were detected by ELISA (ZeptoMetrix, Baffalo, NY, USA; Cat.: 0801111) and diluted to 0.2 ng p24/mL which can be used directly or stored at −80 °C.

Anti-HIV replication activity assay: One day prior to infection, 293T cells were seeded on 24-well plates with the density of 6 × 10^4^ cells per well. Compounds were incubated with cells 15 min ahead of infection. Forty eight h post-infection, infected cells were lysed in 50 *μ*L Cell Lysis Reagent (Promega, San Luis Obispo, CA, USA). Luciferase activity of cell lysate was measured by sirius luminometer (Berthold Detection System, Pforzheim, Germany) according to the manufacturer’s instructions. 

## 4. Conclusions

Two new aromatic compounds, butyl 2,5-dihydroxyphenylacetate (**1**) and butyl 2-O-*β*-D-gluco-pyranosyloxy-5-dihydroxyphenylacetate (**2**), together with three known ones, methyl 2,5-dihydroxyphenylacetate (**3**), ethyl 2,5-dihydroxyphenylacetate (**4**) and 2-O-*β*-D-glucopyranosyloxy-5-hydroxyphenylacetic acid (**5**), were isolated from the kernel of *Entada phaseoloides*. Compounds **1**, **3** and **4** displayed potent inhibitory activities against HIV-1 replication with EC_50_ values of 9.80 *μ*M, 11.70 *μ*M and 9.93 *μ*M, respectively.

## Figures and Tables

**Table 1:** ^1^H- and ^13^C-NMR data of compound **1** and **2** in MeOH-*d*4 (*δ* in ppm, *J* in Hz).

Position	1			2	
	*δ*_H_	*δ*_C_		*δ*_H_	*δ*_C_
1	-	123.8		-	127.7
2	-	150.1		-	150.9
3	6.82 (d, 8.5)	119.1		7.06 (d, 9.0)	119.7
4	6.66 (dd, 8.5, 3.0)	116.2		6.65 (dd, 2.5, 9.0)	116.3
5	-	151.5		-	154.3
6	6.6 1 (d, 8.5)	117.1		6.64 (br s)	118.8
7	3.61 (s)	37.3		3.68 (s)	37.3
8	-	174.8		-	174.9
9	4.14 (t, 6.5)	66.1		4.08 (t, 6.5)	66.4
10	1.64 m	32.3		1.59 m	32.2
11	1.37 m	20.6		1.35 m	20.6
12	0.92 (t, 7.5)	14.5		0.91 (t, 7.5)	14.5
1’	-	-		4.69 (d, 7.5)	104.9
2’	-	-		3.41 (dd, 3.0, 6.0)	75.6
3’	-	-		3.30 m	78.6
4’	-	-		3.39 m	72.0
5’	-	-		3.45 m	78.5
6’	-	-		3.68 (d, 12.5)3.87 (dd, 1.5, 12.5)	63.2
